# How to Manage a Case of Labor

**Published:** 1877-04-20

**Authors:** John W. Martin

**Affiliations:** The Grange, Dronfield, near Sheffield, Late Assistant-Surgeon, Mayfield Factory Dispensary, Portlaw


### HOW TO MANAGE A CASE OF LABOR.

Reported by JOHN W. MARTIN, M. D.,
The Grange, Dronfield, near Sheffield,
Late Assistant-Surgeon, Mayfield Factory Dispensary, Portlaw.

I may perhaps be excused if for the sake of the student, I briefly enumerate the chief points of practical interest to be borne in mind on being called to a case of labor.

1. Be careful to show no consciousness that the occasion is one calling for explanations of what you wish done in the way of examinations, etc. Whatever you have to do, do it quietly and judiciously, carefully and gently, as a mere matter of routine, calling for no comment from either doctor or patient.

2. Ask the patient, if no nurse be in attendance, to lie on her left side, with her hips out well towards you, and her knees well drawn up. If a nurse be present, give her directions to have the patient placed in the proper position.

3. See that your hand is well warmed, and the index finger oiled, or anointed with lard.

4. Previous to making the examination, gain your patient's confidence, and place yourself *en rapport* with her, by asking in a quiet tone a few questions as to the number and nature of her previous confinements, when her labor commenced, etc.

5. Then pass your hand quietly under the bed clothes, and without remark feel gently for the labia, separate them, and avoid the clitoris, pass your finger cautiously and slowly, backwards and upwards, until you reach the cervix uteri.

6. In passing upwards, satisfy yourself as to the state of the rectum, whether loaded with fæces or not.

7. Also as to the coolness and moisture of the vagina, or as to its being hot and dry.

8. Note the general capacity of the pelvic cavity, whether roomy or contracted.

9. Feel for the promontory of the sacrum; if difficult to reach it is a good sign, showing the absence of contracted conjugate diameter, or deformity.

10. Having reached the "cervix," note whether it is pendant into the cavity of the vagina, like the nipple of the breast, or whether it is all taken up, and stretched over the presenting part. If not taken up, and the os remains undilated, it will be additional evidence in favor of concluding that true labor has not yet come on, and that any pains complained of are false pains, to be relieved by a dose of either opium or chloral, or both combined.

11. Quietly make out the position of the os uteri; this is sometimes situated very high up, close to the sacral promontory. Bear in mind the possibility of a double "os" being present, one of which may be dilated, and the other not so. Any oversight on this point might give rise to an obvious error in your prognosis. Note the amount of dilatation, the condition of the edges of the "os," whether thick, rounded, moist, and yielding; or thin, sharp, hard, resisting, and undilatable, and tending to dryness. The latter condition, in the majority of instances threatens a prolonged labor, and usually accompanies a state of debility and want of rest on the part of the patient. In such cases, a good dose of opium or chloral, given as already described, will be found to exert a most beneficial effect, in controlling and properly directing the uterine contractions; the condition of the parts usually change rapidly, the os becoming thick, rounded, and dilatable, and the passages bathed in mucous.

12. Satisfy yourself as to the nature of the presenting part of the fœtus, the condition of the membranes, and the quantity of "fluid" present.

13. It will be found a safe good rule to leave the membranes unruptured to the last; early rupture may lead to troublesome complications. Rupturing at the completion of the first stage sometimes hastens labor; but the risk of inducing a difficult case ought to make it a matter of very careful consideration. I have always found those cases do best in which the membranes remain unruptured, until they appear as a bag, at the external orifice; the descending bag of waters is the natural and best distending force, preparing the way for an easy descent of the head.

14. If the rectum be loaded, clear it out with a simple enema, such as some warm water having a couple of tablespoonfuls of salt and sugar dissolved in it.

15. Learn whether your patient passes water freely; distension of the bladder would prove a source of delay to the birth of the child.

16. If the patient has borne several children, and the abdominal walls are flabby, pendulous, allowing the womb to fall forward, thus altering its proper axis, the application of the binder will be found very useful.

17. Where the pelvis is roomy, the maternal passages are cool, moist and dilatable, the os uteri well dilated and dilatable, the patient passing water freely and the rectum well cleared out. Kristellar's method of assisting labor tedious from inertia, may be found useful. Place the patient on her back: standing above her as it were, apply the palms of both hands to the fundus of the uterus, the fingers and thumbs extending forwards towards the pubis and crest of ilium; then make steady pressure at the onset and during the continuance of each pain, by this means aiding in the expulsion of the child.

18. Do not be in too great a hurry to aid with pressure the exit of the head through the external orifice; give time for the full distention of the perineum, which gradually thins out and expands with each pain; retard the progress of the head if necessary. Attention to this point in my experience reduces the danger of rupture to a minimum, and in the majority of cases renders support to the perineum unnecessary. Of late my experience inclines me to believe, that the application of the hand as a means of support to the perineum is rather injurious than otherwise, heating the part and inducing dryness, thus interfering with its natural distension. The natural course in this instance will very likely prove the best.

19. Upon the child's head being born, clear its mouth and throat of all mucous, by passing your little finger well down into the pharynx; do this cautiously; then examine if the placental cord is twisted round its neck; if it is, pull it out gently, remembering the danger of breaking it, and slip it if possible over the child's head, if not, over its shoulders and body. Support the head whilst awaiting the expulsion of the shoulders and body. During the expulsion of the child, place the left hand upon the fundus of the uterus, and follow it downwards with careful but firm pressure.

20. Upon the birth of the child, again clear out the mouth and throat, and compel it to cry if need be, by slapping it well over the buttocks and back; should any delay occur in setting up of natural respiration, direct the nurse or some friend in attendance to make pressure upon the "fundus uteri," and at once practise artificial respiration, Sylvester's method being the best; apply cold suddenly to the surface of the body and make rapid frictions with either brandy or whisky.

About sixteen respirations to the minute will be sufficient, when practising artificial respiration.

21. Allow a few minutes to pass by before applying the ligatures to the cord to see if pulsation ceases. The ligature next the child's body should be placed some three inches from it, and should be firmly secured with as many as three knots. The ligatures should be made of strong housewife thread, each one being composed of some three or four strands of the thread. After dividing the cord with scissors, dry the surface, and examine if there be any bleeding present. This is a point to be well attended to, as any oversight might lead to injurious effects to the child, and discredit to your own skill and reputation.

22. Allow the womb some fifteen to twenty minutes rest, keeping up firm but gentle pressure over the fundus. Very frequently, at the expiration of the time of grace, the womb expels spontaneously, with a good strong contraction, the placenta and membranes entire.

23. Should delay occur, increase the amount of pressure, apply your hand, previously cooled by being dipped into cold water, suddenly to the surface of the abdomen; these means frequently effect the object you have in view. I would advise, as far as possible, that the cord should not be dragged upon, as is very frequently done and recommended.

24. If firm pressure to the fundus, sudden application of cold to the abdomen, gentle traction, and causing the patient to cough, fail to induce expulsion of the placenta, the right hand must be washed perfectly clean, and then gently introduced per vaginum and the mass removed.

25. Immediately after the delivery of the placenta has taken place, wherever there is the smallest risk of hemorrhage a dose of ergot, half a drachm to two scruples, should be given.

26. A point worth remembering in connection with making pressure upon the fundus uteri is this: do not be satisfied with merely pressing upon the surface of the abdomen, but feel carefully for the body of the womb through the relaxed abdominal walls, and then grasp it firmly, and at the same time gently.

27. In the case of post-partum hemorrhage occurring in addition to the ordinary means for arresting it, viz., pressure, cold applications to the abdomen, administration of ergot in repeated doses, of a half a drachm every ten or fifteen minutes, brandy, elevation of the patient's hips, introduction of the hand into the uterus, mopping out the internal surface of the womb with a solution of the perchloride of iron, one part to six, or the intra-uterine injection of this solution, is recommended; pressure upon the abdominal aorta, the application of the galvanic current, and transfusion may be tried in extreme cases.

2°. Delivery having been effected, the placenta expelled, and no post-partum hemorrhage occuring, all clots of blood must be removed from the bed, the latter being made as dry and comfortable as possible. A clean sheet, in four folds, should be slipped under the hips. If you have taken the precaution before delivery to carry the night-dress well up under the waist, towards the armpits, this will be dry and comfortable. It remains only to apply the binder. This must be done carefully with as little movement of the patient as possible. The binder should be a yard and a half in length, and fifteen inches in breadth; and is best made from a piece of strong old linen or calico. It should be rolled up from one end, to allow of its being easily slipped beneath the patient *en masse.* When binding, place the patient on her left side, with her lower extremities and body in a straight line; place the binder so that its lower border will be below the trochanteric prominences; then fold round the body from below upwards firmly, and bind, securing the bandage, where possible, with safety pins. The upper border need not be drawn very tightly, to prevent its interfering with respiration.

29. If there is any tendency to relaxation on the part of the uterus, a good firm pad may with great advantage be applied over it, beneath the binder.

30. Examine your patient's pulse; if it remains full and firm at 80 beats the minute all is well. If it passes beyond a hundred there is always cause for anxiety and watchfulness.

31. Again examine the portion of cord attached to the child, to be assured that there is no bleeding therefrom. See, also, that the piece of linen in which it is to be wrapped has been well singed at the edges which are to be in immediate contact with the cord.

32. The application of the infant to the breast as soon as dressed, is a safe measure for the mother, tending to prevent hemorrhage.

33. Do not leave the house for an hour after delivery has been effected, and during that time watch carefully the amount of discharge, and the state of contraction on the part of the womb.

34. A thorough acquaintance with the minutiæ of treatment necessary in dealing with ordinary cases, makes it a matter of comparative ease when called upon to treat those which are graver in their nature.—*Med. Press and Cir.*

